# Supplementary material for: Detailed molecular and epigenetic characterization of the pig IPEC-J2 and chicken SL-29 cell lines
Source: iScience. 2023 Feb 20;26(3):106252. doi: 10.1016/j.isci.2023.106252 (PMC10018572; doi:10.1016/j.isci.2023.106252)
Supplement: Data S1. Complete homer output for identified motifs in Pig IPECJ-2, related to Table 2 — Homer motif analysis results for histone modifications H3K4me1, H3K4me3, H3K27ac, and enhancer elements of pig IPECJ2 cell line. P-values >1e-10 are possible false positives. Within each folder (e.g. peak_files_CTCF) are the html files showing the identified motifs when using homer (e.g. homerResults.html). [file mmc2.zip › S5/Pig_IPECJ_2/peak_files_enhancer/homerResults/motif1.similar.html]

motif1

## Information for motif1

A
G
C
T
C
A
T
G
C
G
T
A
A
T
C
G
C
G
A
T
G
T
A
C
T
C
G
A
A
G
C
T
A
G
T
C
A
T
C
G
  
Reverse Opposite:  

T
A
G
C
T
C
A
G
T
C
G
A
A
G
C
T
C
A
T
G
G
C
T
A
T
A
G
C
G
C
A
T
G
T
A
C
T
C
G
A
  

|  |  |
| --- | --- |
| p-value: | 1e-469 |
| log p-value: | -1.080e+03 |
| Information Content per bp: | 1.601 |
| Number of Target Sequences with motif | 4324.0 |
| Percentage of Target Sequences with motif | 11.80% |
| Number of Background Sequences with motif | 2008.8 |
| Percentage of Background Sequences with motif | 5.49% |
| Average Position of motif in Targets | 151.2 +/- 82.9bp |
| Average Position of motif in Background | 150.5 +/- 90.8bp |
| Strand Bias (log2 ratio + to - strand density) | 0.0 |
| Multiplicity (# of sites on avg that occur together) | 1.08 |
| Motif File: | file (matrix) reverse opposite |

### Similar de novo motifs found

|  |  |  |  |  |  |  |  |
| --- | --- | --- | --- | --- | --- | --- | --- |
| Rank | Match Score | Redundant Motif | P-value | log P-value | % of Targets | % of Background | Motif file |
| 1 | 0.959 | C T A G T A C G A C G T A C T G C G T A A T G C A C G T A G T C C G T A A T C G C G T A T C A G G T A C | 1e-419 | -966.817300 | 8.32% | 3.42% | motif file (matrix) |
| 2 | 0.963 | A C G T A C T G C G T A T A G C C G A T G T A C C T G A A G C T | 1e-411 | -946.907697 | 9.80% | 4.42% | motif file (matrix) |
| 3 | 0.847 | C T A G A G C T A C T G C G T A T A G C G A C T C T G A C G T A | 1e-92 | -213.344041 | 6.47% | 4.17% | motif file (matrix) |
| 4 | 0.772 | T A G C C A G T C A T G T C G A A G T C G C A T G T A C G T C A C A T G C T G A | 1e-73 | -169.950171 | 20.43% | 16.77% | motif file (matrix) |
| 5 | 0.639 | A C T G A C G T C G T A C G T A C G A T T C A G C G T A A T C G C G A T G C T A G C T A C A G T A T G C | 1e-43 | -100.258189 | 0.10% | 0.00% | motif file (matrix) |
| 6 | 0.624 | C T A G G C T A C G A T C A G T C G T A C A T G C G A T G T A C G C T A A G T C C G A T C A T G C T A G | 1e-34 | -79.019889 | 0.08% | 0.00% | motif file (matrix) |
| 7 | 0.869 | A G C T A C T G C G T A C G T A A C G T A G T C C G T A A G C T | 1e-32 | -73.790825 | 4.92% | 3.69% | motif file (matrix) |
| 8 | 0.689 | A C T G A G T C A G C T A C G T G T C A A C T G A C G T A G T C C T G A A G T C | 1e-19 | -44.640479 | 0.12% | 0.02% | motif file (matrix) |
